# Comparing Mouse and Human Tissue-Resident γδ T Cells

**DOI:** 10.3389/fimmu.2022.891687

**Published:** 2022-06-08

**Authors:** Guanyu Qu, Shengli Wang, Zhenlong Zhou, Dawei Jiang, Aihua Liao, Jing Luo

**Affiliations:** ^1^ Institute of Reproductive Health, Center for Reproductive Medicine, Tongji Medical College, Huazhong University of Science and Technology, Wuhan, China; ^2^ School of Basic Medicine, Jinan University, Guangzhou, China; ^3^ Institute of Biomedicine and National Engineering Research Center of Genetic Medicine, College of Life Science and Technology, Jinan University, Guangzhou, China; ^4^ Department of Nuclear Medicine, Union Hospital, Tongji Medical College, Huazhong University of Science and Technology, Wuhan, China

**Keywords:** γδ T cells, tissue-resident γδ T cells, human γδ T cells, mouse γδ T cells, γδ T cells development

## Abstract

Circulating immune cell compartments have been extensively studied for decades, but limited access to peripheral tissue and cell yield have hampered our understanding of tissue-based immunity, especially in γδ T cells. γδ T cells are a unique subset of T cells that are rare in secondary lymphoid organs, but enriched in many peripheral tissues including the skin, uterus, and other epithelial tissues. In addition to immune surveillance activities, recent reports have revealed exciting new roles for γδ T cells in homeostatic tissue physiology in mice and humans. It is therefore important to investigate to what extent the developmental rules described using mouse models transfer to human γδ T cells. Besides, it will be necessary to understand the differences in the development and biogenesis of human and mouse γδ T cells; to understand how γδ T cells are maintained in physiological and pathological circumstances within different tissues, as well as characterize the progenitors of different tissue-resident γδ T cells. Here, we summarize current knowledge of the γδ T phenotype in various tissues in mice and humans, describing the similarities and differences of tissue-resident γδ T cells in mice and humans.

## 1 Fundamental Characteristics of γδ T Cells

Gamma delta (γδ) T cells are a small subset of CD3-positive T cells in the peripheral blood but occur at increased frequency in mucosal tissues in mice and humans (1). Murine and human γδ T cells make up a minor part (1–5%) of the circulating T cell compartment found in the blood and secondary lymphoid organs. However, certain subsets of γδ T cells are present in much higher proportions (10–100%) in epithelial tissues, such as the reproductive tract, skin epidermis, and gastrointestinal tract (2). The mouse γδ T cell subsets are distinguished by different T cell receptor(TCR) Vγ chains, whereas human γδ T cell subsets are often distinguished by Vγ chain usage (2).

Heilig and Tonegawa’s nomenclature proposed in 1986 segregated mouse γδ T cells into six distinct subsets: Vγ1, Vγ2, Vγ4, Vγ5, Vγ6, and Vγ7 (3). Meanwhile, the human γ chain locus consists of four subgroups; VγI includes Vγ2, 3, 4, 5, and 8. Among the three other Vγ subgroups, only Vγ9 (from the VγII group) is functional when using the nomenclature of Lefranc and Rabbitts (4). Besides, γδ T cells are reported to bridge the gap between innate and adaptive immune responses in mice and humans. Although γδ bearing cells were shown to constitute a minor proportion of peripheral T lymphocytes, their co-evolution with αβ T cells and B lymphocytes revealed non-redundant functions.

γδ T cells mostly reside within tissues, particularly in epithelial layers, where they might play tissue-protective or inflammatory roles (5). Experiments in mice have demonstrated that γδ T cells are predominantly tissue-resident immune cells (6, 7). From further mouse studies, it is nonetheless becoming increasingly clearer that the γδ T pool residing in a given tissue is the result of the wave of development from fetal to adult life, referred to as layered ontogeny (8). Nevertheless, how the ontogeny of γδ T cells differs between tissues remains obscure. Although the origin of tissue-resident γδ T cells in humans is technically challenging to address, there is evidence that the local γδ T cells pool can partially be replenished by infiltration and *in situ* differentiation of circulating naïve γδ T cells (9).

Reflecting their tissue residency and the impact of the microenvironment on γδ T cell function, recent studies have revealed profound tissue-specific transcriptional signatures for human (9) and mouse γδ T cells (10). Accumulating evidence suggests that γδ T cells are shaped by the microenvironment and exert tissue-specific functions depending on the signals they receive. This review summarizes recent studies on the tissue-specific features of γδ T cells across organs in mice and humans. We discuss the phenotypic differences that contribute to distinct γδ T cell profiles in different tissues, highlighting the similarities and differences between mice and humans. Understanding how various tissue microenvironments impact γδT cells is important for improving therapeutic strategies in pathologies that affect specific tissues.

## 2 γδ T Cells Development in Mice

αβ T cells and γδ T cells arise from a common progenitor known as a double-negative cell (DN; lacking CD4 and CD8 expression) in the thymus (11). γδ T cells that develop without pre-programming in the thymus and receive the TCR signal in the periphery develop as adaptive types, whereas γδ T subsets that receive the signal in the thymus are innate types, and those which receive the TCR signal in the periphery but during an early phase of life get converted into innate-like γδ T cells (12).

**Figure 1:**
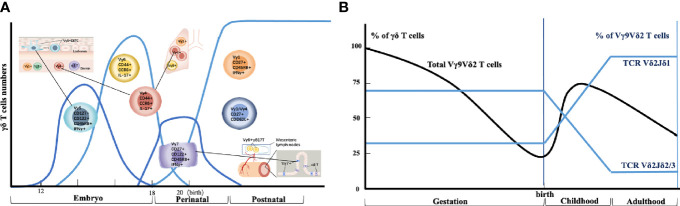
Thymic developmental waves and tissue homing of human and mouse γδ T cell subsets. **(A)** Different waves of γδ T cell progenitor subsets are produced in specific developmental windows in the thymus and selectively home to different organs. **(B)** Schematic depiction of human Vγ9Vδ2^+^ T cell generation and selection throughout life.

During the development of mouse γδ T cells, γδ T cells are the first T cells to develop in the mouse embryonic thymus and appear as early as embryonic day 15 of gestation. These cells express a monoclonal Vγ5Vδ1 T cell receptor (TCR) and are always located in the skin epidermis. A few days later, by an oligoclonal Vγ6Vδ1 TCR-expressing population, entered multiple peripheral locations, including the tongue, dermis, uterus, testis, abdominal cavity, adipose tissue, and meninges. Semi-invariant Vγ4^+^ γδ T cells also develop within this time range, and these cells are associated with Vγ6^+^ cells that have the same functional characteristics. Such Vγ4^+^ γδ T cells are home to the lungs, the dermis of the skin, and lymph nodes. Subsequent perinatal Vγ7^+^ γδ T cell waves enter the intestine, followed by polyclonal Vγ1^+^ and Vγ4^+^ γδ T cell populations, which are more systematically distributed, including peripheral lymphoid organs, where they exhibit adaptive behavior when activated (revised **Figure 1A**).

On the other hand, mouse γδ T cells can commit to effector cytokine production during thymic development; two main functional subsets have been extensively described: IFN-γ-producing γδ T cells and IL-17-producing γδ T cells. (i) IFN-γ-producing γδ T cells express surface markers, such as CD45RB and CD27. Subpopulations include the fetal and perinatally derived Vγ5^+^ dendritic epidermal T cells (which is called DETC), which are home to the skin, and the postnatally generated cells that express more polyclonal γδ T cell receptor (TCRs) (mostly Vγ1^+^ or Vγ4^+^) and localize to lymphoid tissues. (ii) IL-17-producing γδ T cells lack CD27 expression and include the fetal derived monoclonal and/or oligoclonal Vγ6^+^ T cells that are home to the tongue, dermis, uterus, testis, adipose tissue, and brain meninges, and the Vγ4^+^ IL-17-producing γδ T cells that express multiple semi-invariant TCRs and are home to the lung, dermis, and lymph nodes.

The development of mouse γδ T cells and their subsets depends critically on IL-7 and IL-15 (5). The growth of dermal γδ T cells preferentially requires IL-7, whereas IL-15 is mandatory for the generation of γδ TCR-expressing intra-epithelial lymphocytes (IELs) (13). IL-7 signaling promotes the development of IL-17-producing γδ T cells, whereas IL-15 and IL-2 induce IFN-γ secretion. Besides, various cytokines have been reported to affect the differentiation of effector γδ T cells. IL-12 and IL-18 promote IFN-γ production, while IL-1β and IL-23 drive them towards IL-17-producing cells (14).

In summary, these very curious ‘waves’ of mouse γδ T cell development ensure that most peripheral tissues are effectively colonized by long-lived γδ T cells (Revised **Figure 1A**) that are ideally placed to play important roles *in situ*.

## 3 γδ T Cells Development in Humans

Unlike murine γδ T cells, human γδ T cells are usually sub-divided based on the use of one of two variable regions of TCR-δ chains, which is Vδ1 or Vδ2. The Vγ9 and Vδ2 variable (V) gene segments are the first γ/δ chains to undergo rearrangement in development, detected in the fetal liver from as early as at weeks 5~6 of gestation (15) and in the fetal thymus after 8 weeks of gestation (16). By mid-gestation (20~30 weeks), Vγ9Vδ2^+^ T cells dominate the γδ repertoire (Revised **Figure 1B**). Vδ2 is the largest subset of circulating human γδ T cells in the blood, which gets rapidly recruited to the mucosal surface to participate in the clearance of localized infection (17). Functionally, Vδ2^+^ T cells exist as naive (CD45RA^+^CD27^+^), central memory (CD45RA^−^ CD27^+^), effector memory (CD45RA^−^ CD27^−^), and terminally differentiated (CD45RA^+^CD27^−^) populations (18). By contrast, human Vδ1^+^ subsets are the major γδ T cells population in the intestine and skin, whereas Vδ3^+^ subsets are enriched in the liver and gut.

Several features of the Vγ9Vδ2^+^ compartment suggest similarities to mouse γδ T-cell subsets (19). First, the early fetal wave of Vγ9Vδ2^+^ production, with the semi-invariant Vγ9Vδ2^+^ TCR repertoire, mirrors early waves of semi-invariant mouse γδ T cells. Second, the semi-invariant mouse population expresses Vγ4 sequences of restricted length and diversity, analogous to public human Vγ9 sequences (20, 21). Third, consistent with related immunobiology, butyrophilins (BTN3A1 and BTN3A2/3) are important for Vγ9Vδ2^+^ T cell recognition (22). However, while some semi-invariant mouse γδ T cell populations can become hyporesponsive to TCR triggering following initial strong TCR signaling during development (23), apparently, this does not apply to human Vγ9Vδ2^+^ T cells. Notably, Vγ9Vδ2^+^ T cells remain responsive to both pyrophosphate antigens (pAg) and anti-CD3 stimulation, a feature that underlies their potential use in several cancer immunotherapy applications (24), and they also exhibit the potential for further TCR-mediated plasticity (25).

In summary, γδ T cells comprise distinct functional subpopulations. Current views in the field suggest that the functional potential of mouse γδ T cells is related to the use of Vγ, while the functional potential of humans is related to the use of Vδ (26). When assembling TCRs, human γδ T cells express seven bona fide Vγ genes but only three Vδ genes (27).

## 4 Comparison of the Mode of Action of γδ T Cells in Different Anatomical Locations in Mice and Humans

### 4.1 γδ T Cells in the Skin

γδ T cells localized to the skin are mainly involved in maintaining tissue homeostasis and epithelial repair, maintaining epithelial barriers, and contributing to innate immunity. However, the γδ T subsets in mouse and human skin differ.

#### 4.1.1 γδ T Cells in Mouse Skin

The skin is composed of two major compartments, the epidermis and the dermis, that are populated in the steady-state by distinct γδ T cell subsets. Intraepithelial Vγ5^+^ and Vγ6^+^ γδ T cells are present in the dermis (28). In wild-type mice, the epidermal T cell compartment is dominated by a highly specialized γδ T cell subset termed dendritic epidermal T cells (DETCs) (29). DETC precursors that express a canonical Vγ3Vγ1 TCR are the first T cells to develop in the mouse thymus. Vγ3^+^ thymocytes are generated only during the early fetal stages of thymic development from E13 to E18 and migrate to the epidermis, where a defined homeostatic density is maintained throughout life by self-renewal (30). Moreover, SKINT1 was shown to couple thymic selections of DETC precursors to their functional programming as IFN-γ producers (31). SKINT1, a mouse-specific member of the butyrophilins (BTNs) family that is exclusively expressed in the thymic epithelium and the epidermis, was shown to be essential for thymic selection and skin-specific homing of Vγ5Vγ1 T cell (32).

**Figure 2:**
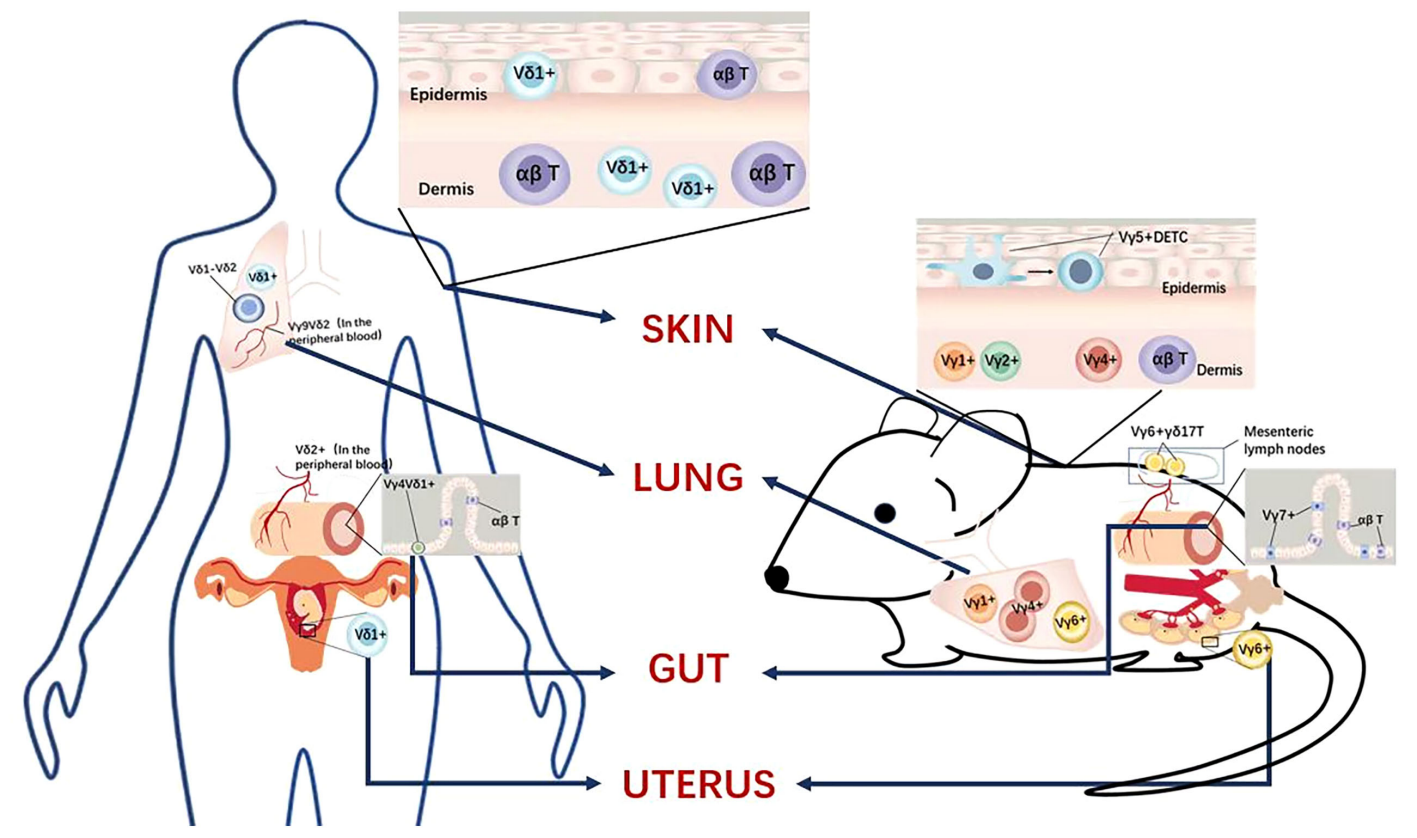
Tissue-resident γδ T cell subsets, comparing humans and mice.

When the skin is damaged or infected, the γδ T cells that function in the epidermis of mouse skin are the epidermis-localized Vγ5^+^ DETCs whose dendritic morphology enables them to contact several adjacent cells simultaneously, such as keratinocytes, Langerhans cells and melanocytes, which increase their own susceptibility to tissue stress and pathology (33). The maintenance of steady-state numbers of DETC is dependent on epithelial cell-derived IL-15, insulin-like growth factor I (IGF1) produced by DETC itself, and through the transcription factor aryl hydrocarbon receptor (AHR) ligand (2). Wendy and colleagues have found that the lack of DETCs in Tcrγ^-/-^ (which means the mice lack all γδ T cell subsets) mice also results in increased keratinocyte apoptosis due to a deficiency of insulin-like growth factor 1 (IGF1) (34). Although DETCs are thymically programmed to produce IFN-γ rather than IL-17 in wild-type mice, DETCs on a skint-1-deficient background are primarily committed toward an IL-17 effector phenotype (35, 36). IL-17 release by DETCs can promote DNA repair following exposure to UV radiation and protect the skin against potential opportunistic infections by releasing keratinocyte-derived antimicrobial peptides (37, 38). However, in the models of psoriasis and dermatitis, IL-17 is detrimental and is produced by dermal Vγ4^+^ and Vγ6^+^ γδ T cells rather than by DETCs. Paradoxically, another study showed that IL-17-producing γδ (γδ17) T cells have a beneficial role in steady-state skin physiology, and γδ17 T cells are also necessary for skin homeostasis (Revised **Figure 2**).

#### 4.1.2 γδ T Cells in Human Skin

The composition of T cell subsets in the skin differs between mice and humans. There is no direct equivalent of DETCs in human skin as the immune cell composition of the epidermis is subject to species-specific differences (2). In human skin, γδ T cells dominate in both the dermis and the epidermis, but γδ T cells are present in both compartments (2).

In humans, the subset of γδ T cells localized in human skin is Vδ1^+^ γδ T cells, which express oligoclonal clonal sequences distinct from circulating γδ T cells (39). Unlike mouse skin epidermal T cells that only contain DETCs, the human epidermis contains both αβ T cells and γδ T cells, and Vδ1^+^ γδ T cells are localized in both the epidermis and dermis (40). Similar to DETCs, human epidermal T cells produce keratinocyte growth factor (KGF) and insulin-like growth factor 1 (IGF1) and promote wound healing upon activation. It can be seen that DETCs can be regarded as a conserved expression in mouse and human skin and have similar functions, but there are differences in the subgroups of γδ in different species. First, the human γδ T cells subsets rarely secrete IL-17, which was quite different from the mouse γδ T cells subset in the skin. Second, the human γδ T cells subset in the skin is Vγ1 γδ T cells verse DETCs in the mouse skin. Third, the mechanism of how human γδ T cell protects from infection is also different from that of the mouse γδ T cells.

In summary, although the role of DETCs in wound healing in mice has been demonstrated, the functions and roles of human epidermal γδ T cells are just beginning to be elucidated (33) (Revised **Figure 2**). There is an urgent need to explore human γδ T cell functions in future work.

### 4.2 γδ T Cells in the Lungs

#### 4.2.1 γδ T Cells in Mouse Lungs

Considerable numbers of Vγ4^+^ and Vγ6^+^ γδ T cells are present in mouse lungs, but their effect on lung tissue physiology is unclear (28). When lung infection occurs, Vγ1^+^, Vγ4^+^, and Vγ6^+^ T cells proliferate in the lung, and Vγ4^+^ γδ T cells secrete CXC chemokine ligand 2 (CXCL2; also known as MIP2) and TNF to promote neutrophil recruitment (41). The secretion of IL-17 by γδ T cells may be the main mechanism involved in lung immunity. Studies have shown that infected dendritic cells, through IL-23, can increase the production of IL-17 by Vγ4^+^ and Vγ6^+^ T cells and promote granuloma formation. IL-17 production by lung-resident Vγ4^+^ T cells can also be increased upon secondary attack (42).

#### 4.2.2 γδ T Cells in Human Lungs

In the human lung, both Vδ1^+^ γδT cells and Vδ2^+^ γδT cells play vital roles. However, the mechanisms of these two subsets in specific diseases and the comparison of the immune effect need further research. During lung infection, Vγ9Vδ2 γδ T cells are aggregated to produce IL-17 and IFN-γ, the former being the most important cytokine in TB protection (43). Vγ9Vδ2 γδ T cells specifically recognize the phosphoantigen (E)-4-hydroxy-3-methylbutylpyrophosphate (HMB-PP), which is abundantly produced by *Mycobacterium tuberculosis*, and this selective immunity elicits rapid and long-lasting memory, rapidly producing more IL-17 and IFN-γ upon pathogen-specific re-challenge, enhancing bacterial clearance (44). In advanced non-small cell lung cancer, Vδ1 γδ T cells and Vδ1-Vδ2-γδ T cells are the main subpopulations of γδ T cells in the lung, and higher levels of intratumoral Vδ1 γδ T cells is a poor prognosis factor (45). Due to the lack of methods to expand Vδ1 γδ T cells in lung cancer *in vitro*, we have not been able to clarify the role of Vδ1 γδ T cells in the lung (46) (Revised **Figure 2**).

### 4.3 γδ T Cells in the Uterus

#### 4.3.1 γδ T Cells in Mouse Uterus

Mouse Vγ6/Vδ1 cells are closely associated with the epithelial tissue of the female reproductive tract and account for a major proportion of γδ T cells in uterine tissue (47). Unlike other subpopulations, Vγ6/Vδ1 cells contain a typical Vγ6 TCR amino acid junction. A recent study has reported that the percentages of γδ T cells were significantly higher in the uterus than in peripheral blood, and most γδ T cells in mouse uterus were distributed in the endometrium (48). Further studies indicated that the majority of γδ T cells in the uterus were memory cells with higher expression of CD44 and CD27 but lower expression of CD62L and CCR7 compared to those in the blood (48). In addition, mouse γδ T cells in the uterus were tissue-resident memory γδ T cells expressing CD69 and expressed high levels of CCR6, GranzymeB, and CD107a. Moreover, γδ T cells in the uterus were activated and fully expressed transcription factor RORγt. After a short time of activation, mouse γδ T cells in the uterus significantly expressed high levels of IL-17 but not IFN-γ, promoting the invasion of murine trophocytes.

#### 4.3.2 γδ T Cells in the Human Uterus

In healthy pregnant women, there was an accumulation of Vδ1^+^ circulating cells, in contrast to women with recurrent abortions where the Vδ2^+^ circulating cells dominated (47). The ratio of activated γδ TCR^+^ cells was significantly increased in normal pregnancies compared to that of recurrent abortions (48). A bias towards circulating Vδ1^+^γδT cells seemed to be required for a successful normal pregnancy. However, the precise role of circulating γδ T cells in pregnancy is not yet completely established. Although convenient to study the γδ T cells subsets during pregnancy in the peripheral blood, it hardly to study that how the circulating Vδ1^+^ cells might simply be a spilling over from the fetus-maternal interface.

## 5 Concluding Remarks

Recent reports have undoubtedly revealed significant tissue-specific functions of γδ T cells. We highlight the distribution, features, and specific markers of distinct subsets of murine and human γδ T cells (Revised **Table 1**). In humans, γδ T cells in blood display a quiescent state and migratory behavior reminiscent of naiüve T cells. By contrast, γδ T cells in peripheral organs make up a spectrum of activation states that differ depending on the organ. Although human γδ T cells deserve more research, mouse γδ T cells display tissue-specific degrees of IFN-γ and IL-17A production that appear to be regulated by factors present in the tissues, such as cytokines. γδ T cells subsets in different organs show variable means of sensing the microenvironment, particularly regarding cytokines. Finally, the tissue-specific functions of γδ T cells, in terms of tissue retention and response to chemokines/cytokines, are not only related to the organ but also to species. Further elucidation of γδ T cell-mediated tissue immunity, particularly in humans, will be necessary to improve the development of tissue-specific immunomodulatory drugs to be used, for example, in inflammatory conditions and cancer.

**Table 1:** Distribution, features and specific markers of distinct subsets of murine and human γδ T cells.

Structural subset	Distribution	Features (mainly cytokines)	specific marker
**Murine γδT Cells**			
** Vγ1**	Lymphoid tissue, liver	IFN-γ, TNFα, IL-4 and IL-17	CD27, CD45RB, CD44, CD122
** Vγ4**	Lymphoid tissue, lung, liver, dermis	IL-17, IFN-γ	CD44, CCR6
** Vγ5** **- DETC**	Epidermis	IFN-γ **- **Sensing skin keratinocyte damage **- **Producing KGF and IGF1 to improve wound healing efficiency and participate in the maintenance of epidermal homeostasis **- **Secreting IL-2, IL-3, granulocyte-macrophage colony-stimulating factor, lymphatic chemokine, etc. to regulate the activation and function of DETCs themselves and keratinocytes and other neighboring cell	CD27, CD44, CD45RB, CD122,
** Vγ6**	Uterus, Lung, tongue, liver etc.	IL-17, IL-22, IFN-γ	CD44, CCR6
** Vγ7**	Intestinal mucosa	IFN-γ	CD27, CD45RB, CD122, CD8α
**Human γδT Cells**			
** Vδ1**	PBMCs, skin, gut, spleen, liver	In epithelium, some functions are similar to DETCs; produce IL-10, a small amount of IL-2, IL-4 and IFN-γ; exhibit cytotoxicity through FasL, perforin, granzyme, etc. **- Subset γδTreg** Mainly secreting IFN-γ and granulocyte-macrophage colony-stimulating factor. Regulating innate and adaptive immune responses to play an important anti-infective role. **- Subset Tγδ17** Expressing granzyme B, FasL, and CD161, but does not produce IL-22 and IFN-γ; in terms of antigen activation, Tγδ17 cells rapidly induce IL-8-mediated migration and phagocytosis of neutrophils, and are IL-dependent -17 Produces beta defensins	NKR, Toll-Like Receptor, CD8
** Vδ2**	PBMCs	- Unique Feature:Activated Vδ2γδ T cells acquire APC properties (such as antigen presentation, co-stimulation and expression of adhesion molecules MHC-II, CD80 and CD86) - As circulating γδ T cells, it also possesses cytotoxicity, cytokine and chemokine production and modulation capabilities against infected or tumor cells	NKG2D, Toll-Like Receptor, CD45
** Vδ3**	PBMCs(very few), Liver	Increasing CD1d recognition and kill CD 1d target cells, releasing Th1, Th2 and Th17 cytokines, and inducing dendritic cells to become APCs, when stimulated by mitogens and IL-2.	CD56, CD161, HLA-DR, NKG2D

## Author Contributions

GQ and SW helped in drafting the manuscript. ZZ and DJ helped draw the image in **Figures 1**, **2**. JL and AL conceptualized and revised the manuscript. All authors contributed to the article and approved the submitted version.

## Funding

This work was supported by a grant from the National Natural Science Foundation of China (31900657 to JL).

## Conflict of Interest

The authors declare that the research was conducted in the absence of any commercial or financial relationships that could be construed as a potential conflict of interest.

## Publisher’s Note

All claims expressed in this article are solely those of the authors and do not necessarily represent those of their affiliated organizations, or those of the publisher, the editors and the reviewers. Any product that may be evaluated in this article, or claim that may be made by its manufacturer, is not guaranteed or endorsed by the publisher.

## References

[1] Kabelitz D. Gamma Delta T Cells (Gamma Delta T Cells) in Health and Disease: In Memory of Professor Wendy Havran. Cells (2020) 9(12):2564. doi: 10.3390/cells9122564 PMC7760329 33266147

[2] Nielsen MM Witherden DA Havran WL. Gamma Delta T Cells in Homeostasis and Host Defence of Epithelial Barrier Tissues. Nat Rev Immunol (2017) 17(12):733–45. doi: 10.1038/nri.2017.101 PMC5771804 28920588

[3] Heilig JS Tonegawa S. Diversity of Murine Gamma Genes and Expression in Fetal and Adult T Lymphocytes. Nature (1986) 322(6082):836–40. doi: 10.1038/322836a0 2943999

[4] Lefranc MP Rabbitts TH. A Nomenclature to Fit the Organization of the Human T-Cell Receptor Gamma and Delta Genes. Res Immunol (1990) 141(7):615–8. doi: 10.1016/0923-2494(90)90068-a 2151348

[5] Giri S Lal G. Differentiation and Functional Plasticity of Gamma-Delta (γδ) T Cells Under Homeostatic and Disease Conditions. Mol Immunol (2021) 136:138–49. doi: 10.1016/j.molimm.2021.06.006 34146759

[6] Cheng M Hu S. Lung-Resident γδ T Cells and Their Roles in Lung Diseases. Immunology (2017) 151(4):375–84. doi: 10.1111/imm.12764 PMC5506441 28555812

[7] Bonneville M O'Brien RL Born WK. Gammadelta T Cell Effector Functions: A Blend of Innate Programming and Acquired Plasticity. Nat Rev Immunol (2010) 10(7):467–78. doi: 10.1038/nri2781 20539306

[8] Vantourout P Hayday A. Six-Of-the-Best: Unique Contributions of γδ T Cells to Immunology. Nat Rev Immunol (2013) 13(2):88–100. doi: 10.1038/nri3384 23348415 PMC3951794

[9] Pang DJ Neves JF Sumaria N Pennington DJ. Understanding the Complexity of γδ T-Cell Subsets in Mouse and Human. Immunology (2012) 136(3):283–90. doi: 10.1111/j.1365-2567 PMC3385028 22385416

[10] Ribot JC Lopes N Silva-Santos B. γδ T Cells in Tissue Physiology and Surveillance. Nat Rev Immunol (2021) 21(4):221–32. doi: 10.1038/s41577-020-00452-4 33057185

[11] Koch U Fiorini E Benedito R Besseyrias V Schuster-Gossler K Pierres M. Delta-Like 4 Is the Essential, Nonredundant Ligand for Notch1 During Thymic T Cell Lineage Commitment. J Exp Med (2008) 205(11):2515–23. doi: 10.1084/jem.20080829 PMC2571927 18824585

[12] Parker ME Ciofani M. Regulation of γδ T Cell Effector Diversification in the Thymus. Front Immunol (2020) 11:42. doi: 10.3389/fimmu.2020.00042 32038664 PMC6992645

[13] Dunne MR Byrne G Chirdo FG Feighery C. Coeliac Disease Pathogenesis: The Uncertainties of a Well-Known Immune Mediated Disorder. Front Immunol (2020) 8:1374(11). doi: 10.3389/fimmu.2020.01374 PMC7360848 32733456

[14] Ribeiro ST Ribot JC Silva-Santos B. Five Layers of Receptor Signaling in γδ T-Cell Differentiation and Activation. Front Immunol (2015) 6:15. doi: 10.3389/fimmu.2015.00015 25674089 PMC4306313

[15] McVay LD Carding SR. Extrathymic Origin of Human Gamma Delta T Cells During Fetal Development. J Immunol (1996) 157(7):2873–82. doi: 10.1084/jem.184.4.1585 8816392

[16] McVay LD Jaswal SS Kennedy C Hayday A Carding SR. The Generation of Human Gammadelta T Cell Repertoires During Fetal Development. J Immunol (1998) 160(12):5851–60. 9637496

[17] McCarthy NE Eberl M. Human γδ T-Cell Control of Mucosal Immunity and Inflammation. Front Immunol (2018) 9:985. doi: 10.3389/fimmu.2018.00985 PMC5949325 29867962

[18] Fichtner AS Ravens S Prinz I. Human γδ TCR Repertoires in Health and Disease. Cells (2020) 9(4):800. doi: 10.3390/cells9040800 PMC7226320 32225004

[19] Blazquez JL Benyamine A Pasero C Olive D. New Insights Into the Regulation of γδ T Cells by BTN3A and Other BTN/BTNL in Tumor Immunity. Front Immunol (2018) 9:1601. doi: 10.3389/fimmu.2018.01601 30050536 PMC6050389

[20] Kashani E Föhse L Raha S Sandrock I Oberdörfer L Koenecke C. A Clonotypic Vγ4jγ1/Vδ5dδ2jδ1 Innate γδ T-Cell Population Restricted to the CCR6^+^CD27^−^Subset. Nat Commun (2015) 6:6477. doi: 10.1038/ncomms7477 25765849

[21] Kabelitz D Déchanet-Merville J. Recent Advances in Gamma/Delta T Cell Biology: New Ligands, New Functions, and New Translational Perspectives. Front Immunol (2015) 6:371. doi: 10.3389/fimmu.2015.00371 26257738 PMC4508528

[22] Vantourout P Laing A Woodward MJ Zlatareva I Apolonia L Jones AW. Heteromeric Interactions Regulate Butyrophilin (BTN) and BTN-Like Molecules Governing γδ T Cell Biology. Proc Natl Acad Sci USA (2018) 115(5):1039–44. doi: 10.1073/pnas.1701237115 PMC5798315 29339503

[23] Wencker M Turchinovich G Di Marco Barros R Deban L Jandke A Cope A. Innate-Like T Cells Straddle Innate and Adaptive Immunity by Altering Antigen-Receptor Responsiveness. Nat Immunol (2014) 15(1):80–7. doi: 10.1038/ni.2773 PMC6485477 24241693

[24] Herrmann T Fichtner AS Karunakaran MM. An Update on the Molecular Basis of Phosphoantigen Recognition by Vγ9vδ2 T Cells. Cells (2020) 9(6):1433. doi: 10.3390/cells9061433 PMC7348870 32527033

[25] Imbert C Olive D. γδ T Cells in Tumor Microenvironment. Adv Exp Med Biol (2020) 1273:91–104. doi: 10.1007/978-3-030-49270-0_5 33119877

[26] Prinz I Silva-Santos B Daniel J. Functional Development of γδ T Cells. Eur J Immunol (2013) 43(8):1988–94. doi: 10.1002/eji.201343759 23928962

[27] Adams K the V. D, and J Gene Segments Used in the Primate γδ T-Cell Receptor Reveals a Dichotomy of Conservation and Diversity. Proc Natl Acad Sci U.S.A. (2011) 108(29):11743–4. doi: 10.1073/pnas.1105105108 PMC3141992 21730193

[28] Ribot JC Lopes N Silva-Santos B. Gamma Delta T Cells in Tissue Physiology and Surveillance. Nat Rev Immunol (2021) 21(4):221–32. doi: 10.1038/s41577-020-00452-4 33057185

[29] Xu Y Dimitrion P Cvetkovski S Zhou L Mi QS. Epidermal Resident γδ T Cell Development and Function in Skin. Cell Mol Life Sci (2021) 78(2):573–80. doi: 10.1007/s00018-020-03613-9 PMC11073445 32803399

[30] Takagaki Y DeCloux A Bonneville M Tonegawa S. Diversity of Gamma Delta T-Cell Receptors on Murine Intestinal Intra-Epithelial Lymphocytes. Nature (1989) 339(6227):712–4. doi: 10.1038/339712a0 2544806

[31] Xiang J Qiu M Zhang H. Role of Dendritic Epidermal T Cells in Cutaneous Carcinoma. Front Immunol (2020) 11:1266. doi: 10.3389/fimmu.2020.01266 PMC7381160 32765487

[32] Sutoh Y Mohamed RH Kasahara M. Origin and Evolution of Dendritic Epidermal T Cells. Front Immunol (2018) 9:1059. doi: 10.3389/fimmu.2018.01059 PMC5960712 29868019

[33] Chodaczek G Papanna V Zal MA Zal T. Body-Barrier Surveillance by Epidermal γδ TCRs. Nat Immunol (2012) 13(3):272–82. doi: 10.1038/ni.2240 PMC3288780 22327568

[34] Sharp LL Jameson JM Cauvi G Havran WL. Dendritic Epidermal T Cells Regulate Skin Homeostasis Through Local Production of Insulin-Like Growth Factor 1. Nat Immunol (2005) 6(1):73–9. doi: 10.1038/ni1152 15592472

[35] MacLeod AS Hemmers S Garijo O Chabod M Mowen K Witherden DA. Dendritic Epidermal T Cells Regulate Skin Antimicrobial Barrier Function. J Clin Invest (2013) 123(10):4364–74. doi: 10.1172/JCI70064 PMC3784546 24051381

[36] MacLeod AS Rudolph R Corriden R Ye I Garijo O Havran WL. Skin-Resident T Cells Sense Ultraviolet Radiation-Induced Injury and Contribute to DNA Repair. J Immunol (2014) 192(12):5695–702. doi: 10.4049/jimmunol PMC4048764 24808367

[37] Holtmeier V Pfander M Hennemann A Zollner TM Kaufmann R Caspary WF. The TCR Delta Repertoire in Normal Human Skin is Restricted and Distinct From the TCR Delta Repertoire in the Peripheral Blood. J Invest Dermatol (2001) 116(2):275–80. doi: 10.1046/j.1523-1747.2001.01250.x 11180004

[38] Daniels J Doukas PG Escala MEM Ringbloom KG Shih DJH Jingyi Y. Cellular Origins and Genetic Landscape of Cutaneous Gamma Delta T Cell Lymphomas. Nat Commun (2020) 11(1):1806. doi: 10.1038/s41467-020-15572-7 32286303 PMC7156460

[39] Nakasone C Yamamoto N Nakamatsu M Kinjo T Miyagi K Uezu K. Accumulation of Gamma/Delta T Cells in the Lungs and Their Roles in Neutrophil-Mediated Host Defense Against Pneumococcal Infection. Microbes Infect (2007) 9(3):251–8. doi: 10.1016/j.micinf.2006.11.015 17306586

[40] Lockhart E Green AM Flynn JL. IL-17 Production is Dominated by Gammadelta T Cells Rather Than CD4 T Cells During Mycobacterium Tuberculosis Infection. J Immunol (2006) 177(7):4662–9. doi: 10.4049/jimmunol.177.7.4662 16982905

[41] Shen H Wang Y Chen CY Frencher J Huang D Yang E. Th17-Related Cytokines Contribute to Recall-Like Expansion/Effector Function of HMBPP-Specific Vγ2vδ2 T Cells After Mycobacterium Tuberculosis Infection or Vaccination. Eur J Immunol (2015) 45(2):442–51. doi: 10.1002/eji.201444635 PMC4916493 25141829

[42] Misiak A Wilk MM Raverdeau M Mills K. IL-17–Producing Innate and Pathogen-Specific Tissue Resident Memory γδ T Cells Expand in the Lungs of Bordetella Pertussis–Infected Mice. J Immunol (2017) 198(1):363–74. doi: 10.4049/jimmunol.1601024 27864475

[43] Dieli F Stassi G Todaro M Meraviglia S Caccamo N Cordova A. Distribution, Function and Predictive Value of Tumor-Infiltrating γδ T Lymphocytes. Oncoimmunology (2013) 2(4):e23434. doi: 10.4161/onci.23434 23734305 PMC3654575

[44] Bao Y Guo L Mo J. Characterization of γδ T Cells in Patients With non-Small Cell Lung Cancer. Oncol Lett (2017) 14(1):1133–40. doi: 10.3892/ol.2017.6191 PMC5494795 28693285

[45] Crawford G Ray A Gudi A Shah A Homburg R. The Role of Seminal Plasma for Improved Outcomes During *In Vitro* Fertilization Treatment: Review of the Literature and Meta-Analysis. Hum Reprod Update (2015) 21(2):275–84. doi: 10.1093/humupd/dmu052 25281684

[46] Kang S Wu Q Huang J Yang B Liang C Chi P. Tissue Resident Memory γδt Cells in Murine Uterus Expressed High Levels of IL-17 Promoting the Invasion of Trophocytes. Front Immunol (2021) 11:588227. doi: 10.3389/fimmu.2020.588227 33519808 PMC7840782

[47] Mincheva-Nilsson L. Pregnancy and Gamma/Delta T Cells: Taking on the Hard Questions. Reprod Biol Endocrinol (2003) 1:120. doi: 10.1186/1477-7827-1-120 14651751 PMC305336

[48] Szekeres-Bartho J Barakonyi A Miko E Polgar B Palkovics T. The Role of Gamma/Delta T Cells in the Feto-Maternal Relationship. Semin Immunol (2001) 13(4):229–33. doi: 10.1006/smim.2000.0318 11437630

